# Case Report: Early Transplant Rejection of a Methanol-Intoxicated Donor Heart in a Young Female Patient. A Diagnostic Approach With CMR, Cardiac Biopsy, and Genetic Risk Assessment

**DOI:** 10.3389/fimmu.2020.575635

**Published:** 2021-02-22

**Authors:** Lukas Stoiber, Felix Schoenrath, Christoph Knosalla, Hendrik Milting, Karin Klingel, Carsten Tschöpe, Radu Tanacli, Rolf Gebker, Alexander Berger, Burkert Pieske, Sebastian Kelle

**Affiliations:** ^1^ Department of Cardiothoracic and Vascular Surgery, German Heart Center Berlin, Berlin, Germany; ^2^ Department of Internal Medicine and Cardiology, German Heart Center Berlin, Berlin, Germany; ^3^ DZHK (German Center for Cardiovascular Research), Partner Site Berlin, Berlin, Germany; ^4^ Erich and Hanna Klessmann Institute for Cardiovascular Research & Development (EHKI), Heart and Diabetes Center North Rine-Westphalia (NRW), University Hospital of the Ruhr-University Bochum, Bad Oeynhausen, Germany; ^5^ Cardiopathology, Institute for Pathology and Neuropathology, University Hospital Tübingen, Tübingen, Germany; ^6^ Department of Internal Medicine and Cardiology, Charité University Medicine Berlin, Berlin, Germany

**Keywords:** cardiac magnetic resonance imaging (CMR), heart transplantation, gene sequence, cardiomyopathy, mapping - magnetic resonance imaging

## Abstract

This case report describes the contributions of multimodality imaging, cardiac biopsy, and genetic sequencing to the diagnosis and management of heart transplant rejection in a 23-year old patient with dilated cardiomyopathy.

## Introduction

Graft rejection represents a major factor of limited survival after solid organ transplantation. In patients receiving heart transplants, T-lymphocyte mediated cellular rejection is observed most frequently, followed by antibody-mediated rejection (AMR) with activation of the complement cascade (1, 2). Today, the presence of donor-specific anti-human leukocyte antigen (HLA) antibodies is known to be essential for allograft rejection after heart transplant (3, 4). AMR was recognized first in heart allografts by E. Hammond and co-workers in 1989 (5). In 2013, a modified nomenclature with diagnostic criteria and a unified reporting system was developed for heart transplantation (4, 6). Histopathological staining from cardiac biopsy represents the gold standard for evaluation of possible rejection and helps to assess potential treatment effects. However, the effectiveness of treatment in AMR remains uncertain, and there is a lack of prospective studies addressing this issue. The potential use of gene expression profiling to correctly diagnose AMR and adequately custom therapy is under current investigation (7).

In the present article, we report on a today 25-year old heart recipient with the clinical picture of AMR in the early phase after transplant. We describe therapeutic and diagnostic features including gene encoding sequences for cardiomyopathies and discuss its use in the context of early allograft dysfunction.

## Case Report

A today 25-year old woman with a complex 3-year history of cardiomyopathy following viral myocarditis underwent successful orthotopic heart transplantation at our institution.

Six months prior to transplantation, she was listed in a prioritized status (Eurotransplant “HU” high urgent) but heart failure symptoms worsened with refractory signs of cardiogenic shock despite increased inotropic support. INTERMACS (Interagency Registry for Mechanically Assisted Circulatory Support) profile was at level 2. Left ventricular assist device (LVAD, HeartWare, HeartWare Inc.) was implanted in April 2017, i.e. 6 months before transplantation. During the initial postoperative period, the patient developed treatment-refractory right heart failure. Weaning of inotropic agents was again unsuccessful and led to massive dizziness, arrhythmia, and impaired flow of the LVAD. The patient was again placed in the highest priority status (Eurotransplant “HU” high urgent) for a heart transplant while staying hospitalized in the ICU. Inotropic support before transplantation was maintained with Dobutamine (3.2 µg/kg/min) and Milrinone (0.5 µg/kg/min). LVAD flow remained around 3.8–4.1 l/min.

At the time of admission for transplantation, the patient had well-controlled pulmonary pressure (18/13 mm Hg) and low PVR (95 Dynes.s.cm^-5^). ABO status was compatible (B-/B-), cytomegalovirus mismatched (CMV −/+). The initial HLA antibody results of this mother were reported as being positive for Class I (Luminex Screen solid-phase arrays) specified for HLA B13, B41, B44, B45, B47, B49, B50, B60, B62, B71, B72, B76, and in a weaker expression for HLA B51, B54, B55, B56, B59, B63, B78. No preformed antibodies were cytotoxic. No C1q binding ability could be detected before transplant. The prospective donor-specific crossmatch was negative.

The donor was a 51-year old woman with a history of moderate alcohol consumption and chronic pancreatitis who was transferred to the emergency department of another hospital after the intentional ingestion of Methanol. Despite vasopressor support with noradrenaline, continuous hemodialysis, and therapy directed towards the preservation of neuronal function the donor’s condition had evolved to brain death four days after admission. Echocardiography revealed normal heart function and diameters. Cardiac catheterization ruled out coronary artery disease, and the ECG was unremarkable.

The heart transplantation was uneventful with an allograft ischemic time of 287 minutes. The LVAD was explanted. Pathological findings of the native heart confirmed interstitial fibrosis and mild hypertrophy consistent with the etiology of dilated postmyocarditic heart disease. The patient received induction therapy with cumulative 2 mg/kg body weight (120 mg) of anti-thymocyte globulin (ATG) and triple‐maintenance immunosuppression therapy with Cyclosporine (switched to Tacrolimus on POD 9), Mycophenolate Mofetil, and Prednisone. The retrospective HLA crossmatch was negative; the retrospective amount of HLA mismatch was HLA-A/B/C/DR/DQ: 0/2/-/2/2. The initial postoperative course was uncomplicated, and the patient was extubated 48 hours after surgery.

On postoperative day (POD) 5, a decrease in myocardial function was noticed on transthoracic echocardiographic (TTE) follow up (FU). Pulsed-wave tissue Doppler imaging (PW-TDI) revealed a reduction of the radial and longitudinal systolic peak velocities (Sm) from 10/9 cm/s to 6/8 cm/s as signs for potential rejection (8). At this time point, the patient was already off inotropic support. The ECG remained unchanged (9). Right ventricular (RV) biopsy was performed. Histology revealed no cellular rejection (Level 0R of the International Society for Heart and Lung Transplantation criteria, IHSLT), but acute pathologic antibody-mediated rejection (pAMR grade 1 (I+), i.e. positive immunohistochemical staining with normal biopsy history.

We administered 60 mg of ATG, two infusions with low‐dose intravenous immunoglobulin (1mg/kg body weight each), combined with steroid pulse therapy. No plasmapheresis was performed. At this time point, the expression of the donor-specific HLA Class 1 IgG against B44 had increased and C1q binding ability could be detected for non-donor specific B45 with a possible crossmatch. Six hours after RV biopsy, the patient presented signs of hemorrhagic shock. Computer tomography revealed acute retroperitoneal bleeding as a complication of the femoral puncture and the patient had to undergo emergent surgical decompression. Transfusion of 2 units of ABO-compatible packed red blood cells was necessary. The postoperative course was satisfying, with an echocardiogram showing ongoing improvement in cardiac function (PW-TDI Sm 10/9 cm/s). Ongoing tests on donor-specific HLA-IgG-antibodies revealed increased activity against B44, and increased complement binding activity of these antibodies was detected for the first time on POD 11. The patient was transferred to the transplantation unit of our institution on POD 12. Four cycles of plasmapheresis were performed. Two more doses of 60 mg ATG were administered when a slight decrease in myocardial function was again observed on POD 13. Despite a first remission in the histological stains on POD 21 (pAMR grade 0 in second RV biopsy), the patient presented persistent atrial arrhythmias (atrial flutter with varying A-V conduction) as well as non-sustained ventricular tachycardia (nsVT).

**Figure 1:**
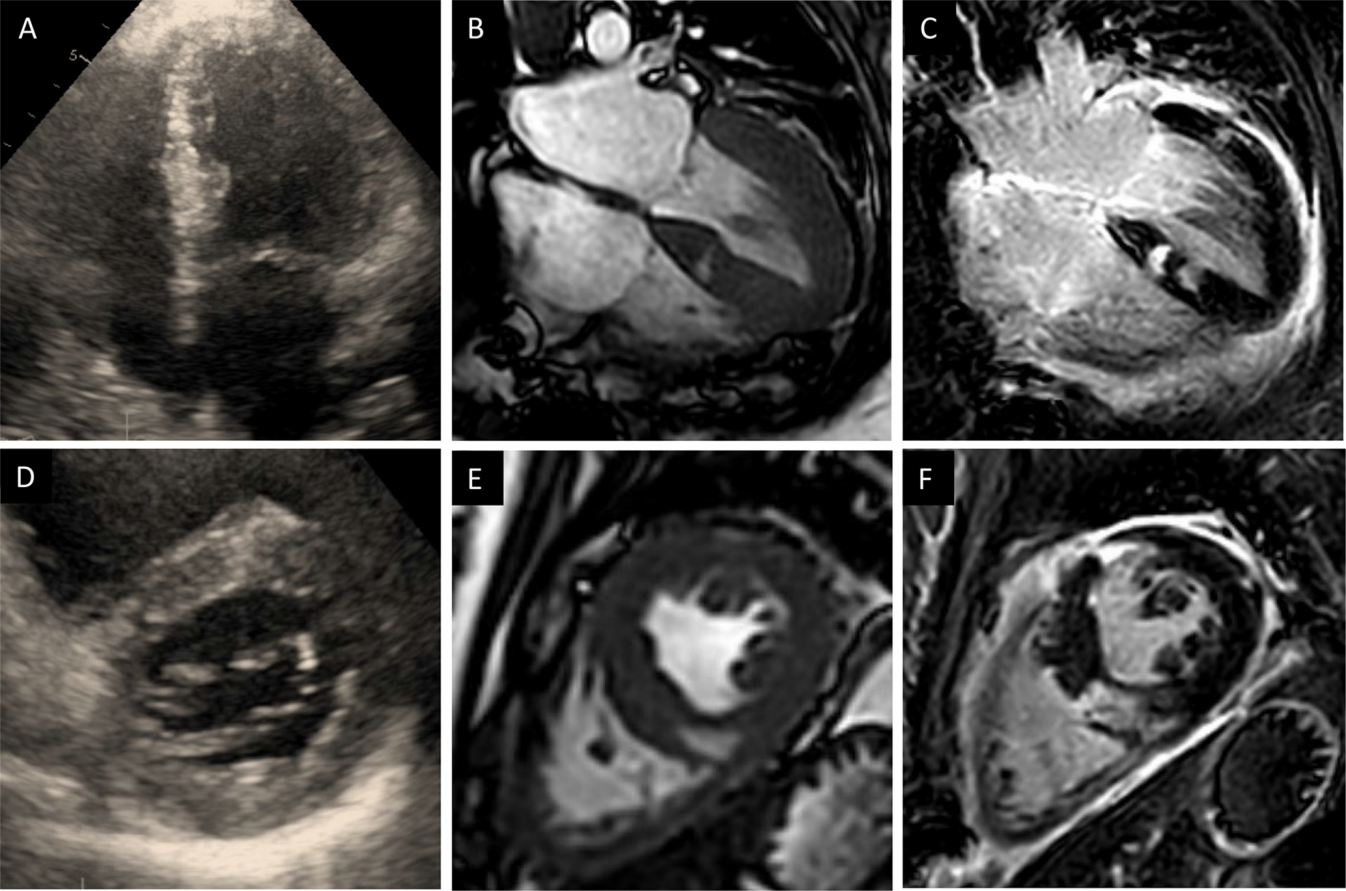
**(A–C)**, Four-chamber transthoracic echocardiography **(A)** and Cardiac magnetic resonance (CMR) images **(B)** reveal concentric hypertrophy with moderately impaired left ventricular function and multiple cystic structures in the septal, mediolateral and anterior parts of the left ventricle. **(C)** Late gadolinium enhancement (LGE) demonstrated high signal intensity of the cystic lesions indicating fibrotic tissue **(D–F)**, Short axes of the corresponding sequences: transthoracic echocardiography **(D)**, Cine-CMR **(E)** and LGE **(F)**.

A first CMR was performed on a Philips Ingenia 3.0 Tesla Scanner one month after transplantation (**Figure 1**). The exam revealed concentric left ventricular (LV) hypertrophy with moderately impaired biventricular function in line with prior TTE findings. Multiple cystic structures were found in the septal, mediolateral, and anterior parts of the LV with a crescent-shaped course ranging from the cavum through the myocardium to the epicardium. Late Gadolinium Enhancement (LGE) demonstrated high signal intensity of the described cystic lesions indicating fibrotic replacement. There was no evidence of regional myocardial edema in the T2-weighted images outside the above areas. Myocardial relaxation times were slightly increased (T1 native 1,389 ms; normal range 1,098–1,354 ms at 3 Tesla. T2 52 ms; normal range 35–51 ms at 3 Tesla).

RV biopsy four weeks after transplant revealed ongoing AMR (pAMR grade 2) with T-cell infiltrates and cell destruction including the vessels. Three more cycles of plasmapheresis, three doses of ATG, and steroid pulse therapy were administered. Five days later, a single dose of Rituximab was given to the patient.

**Figure 2:**
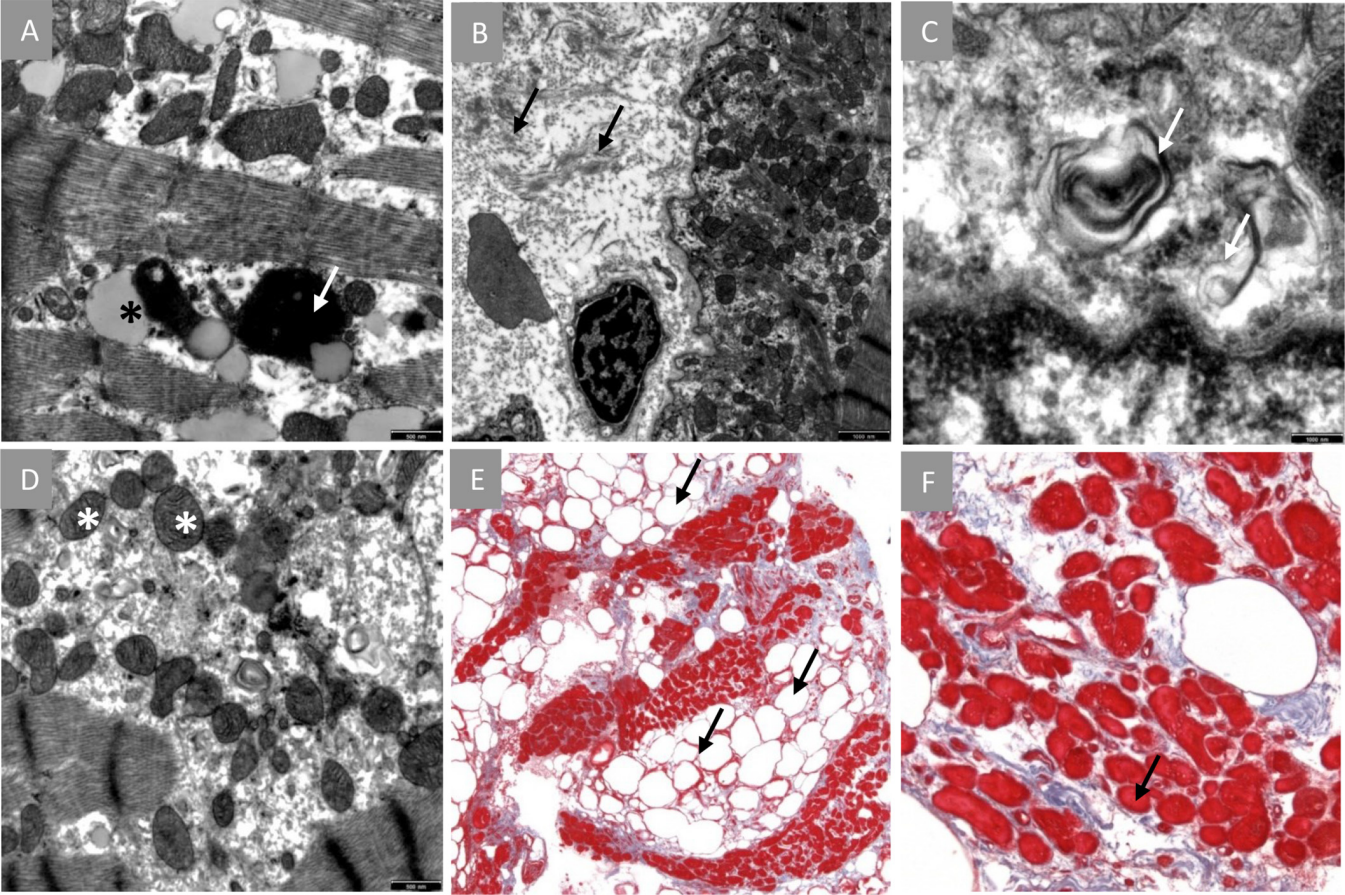
Endomyocardial biopsy findings. **(A–D)**, Electron microscopy: **(A)**, intracellular presence of fatty infiltrations (black star) and degenerative products (white arrow). **(B)**, presence of numerous interstitial collagen fibrils. **(C)**, degenerative structures non-typical for Fabry’s disease. **(D)**, normal mitochondria. **(E, F)**, Masson’s trichome stain: **(E),** high extent of interstitial fibrosis (blue tissue) and abnormal fatty infiltration (arrows). **(F)**, Cardiomyocytes show dystrophic changes (indicated myocyte diameter 11 µm).

In the 6-week FU, CMR LV-function had increased to about 50–55%. The cystic structures in the LV remained unchanged. Corresponding T2 images were strongly hyperintense and the LGE pattern was suspicious for mitochondrial cardiomyopathy. Electron microscopy of the biopsy specimens was performed. Ultrastructural findings revealed the presence of intracellular fat droplets, a high amount of fibrous tissue, and degenerative products (**Figure 2**). The morphology of mitochondria was found to be normal. The high amount of fatty-fibrous tissue in the right ventricle raised the suspicion of Arrhythmogenic right ventricular cardiomyopathy (ARVC) or another specific underlying cardiac disease of the donor.

A genetic study of donor DNA was initiated. Next-generation sequencing with Illumina TruSight™ Cardio Sequencing Kit (Illumina, San Diego, CA, USA) including 174 potential genes for Cardiomyopathies was performed. A detailed list of investigated genes can be found in the **Supplementary Material**.

Sequencing revealed two genetic variants in the coding gene for Titin with uncertain impact on the pathogenesis of ARVC, i.e. classification 3 in the American College of Medical Genetics and Genomics (ACMG) guidelines (**Table 1**). No other specific findings, i.e. no genetic variants explaining either the persistent rejection or the cystic changes could be detected.

**Table 1:** Genetic mutations found in Next-generation sequencing with Illumina TruSight™ Cardio Sequencing Kit.

Gene	Reference-sequence	Genotype/Phenotype	Variant/Mutation	ACMG^1^ classification
Titin (TTN)	NM_001267550	OMIM^2^-G 188840/ OMIM^2^-P 604145	p.Thr20167Ser (c.60500C>G) heterozygous	3
Titin (TTN)	NM_001267550	OMIM^2^-G 188840/ OMIM^2^-P 604145	p.Arg27839Gln (c.83516G>A) heterozygous	3

^1^ACMG, American College of Medical Genetics and Genomics ^2^ OMIM, online mendelian inheritance of men.

One hundred seventy-four potential genes for cardiomyopathies were investigated.

FU TTE found levels of Sm to remain on a relatively low level despite the treatment efforts (10/8 cm/s for radial and longitudinal values respectively). Expression of the donor-specific HLA Class 1 antibodies against B44 was still detectable at week seven after transplant. Steroid pulse therapy, three more cycles of plasmapheresis (without additional ATG) plus increased dosages of mycophenolate mofetil (4g/d) were administered. Under this regimen, the patient finally attained and maintained remission. RV biopsy at week eight after transplant showed no signs of cellular or antibody-mediated rejection (ISHLT level 0R, pAMR grade 0). Despite the high frequency of intravenous steroids and the required changes in the immunosuppressive regimen, the transplant course was uneventful regarding infection. The patient’s tacrolimus levels were maintained at around 12–14 ng/ml.

**Figure 3:**
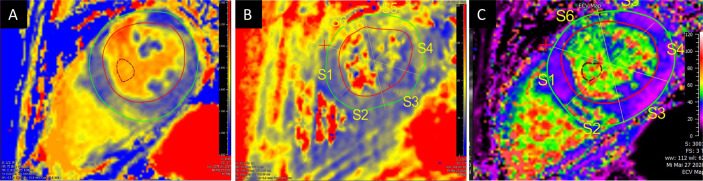
Cardiovascular magnetic resonance mapping of T1 **(A)**, T2 **(B)** and extracellular volume **(C)** at 32 months post-transplant FU revealed increased values. A: T1 Mapping (1425 ± 144); B: T2 Mapping (60.5 ± 8.2); C: Extracellular volume (32.7 ± 9.6).

**Figure 4:**
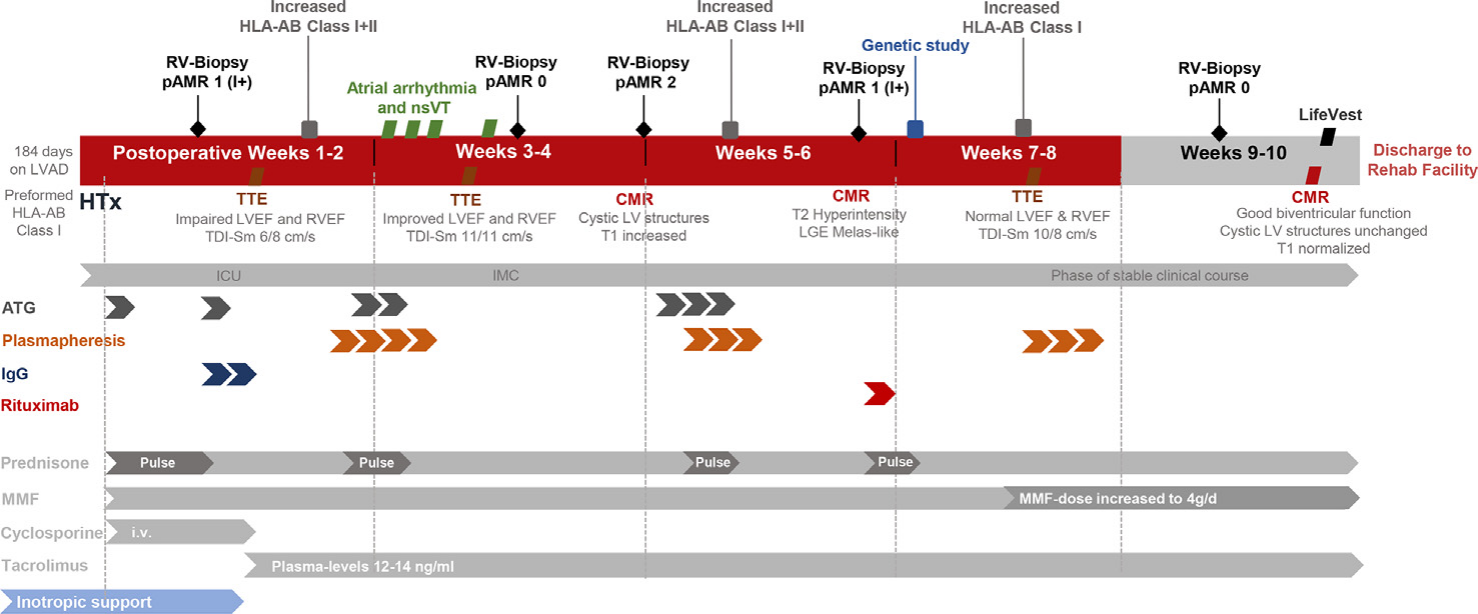
Timeline of postoperative course after heart transplantation. ATG, Antithymocyte globulin; CMR, Cardiac magnetic resonance tomography; HLA-AB, Human leukocyte antigen antibodies; ICU, Intensive care unit; IgG, Immunoglobulin G; IMC, Intermediate care unit; LV, Left Ventricle; LVEF, Left ventricular ejection fraction; LVAD, Left ventricular assist device; MELAS, Mitochondrial encephalomyopathy, lactic acidosis and stroke-like episodes; MMF, Mycophenolate Mofetil; nsVT, Non sustained ventricular tachycardia: pAMR, Pathologic Antibody mediated rejection; RV, Right ventricle; RVEF, Right ventricular ejection fraction; TDI-Sm, Tissue Doppler Imaging Systolic peak velocities (radial/longitudinal); TTE, Transthoracic echocardiography.

Before discharge CMR before discharge showed normal T1 values as compared to the previous examinations as a possible sign of remission. No change in the intramyocardial cysts was found. A wearable cardioverter defibrillator (LifeVest, ZOLL, Pittsburgh, PA, USA) was put on to prevent cardiac arrest due to heart rhythm disturbances. The patient was discharged ten weeks after transplantation to a cardiovascular rehabilitation center. TTE, lab, and clinical state remained stable, and the patient was in a very satisfying condition when seen in the outpatient department for regular clinical, biochemical, and echocardiographic FU. RV biopsy FU exams showed no pathology for cellular or humoral rejection. At the time of writing this report, 32 months after transplant have elapsed and the patient is in a stable clinical state. CMR 32 months FU shows cardiac function to remain stable. However, measures of extracellular volume remain slightly elevated (ECV 33%, cutoff 30%) indicating diffuse fibrosis of the myocardium (**Figure 3**). The described postoperative course is summarized as a Timeline in **Figure 4**.

## Discussion

Our case illustrates the clinical picture of a young woman who underwent orthotopic heart transplantation after suffering from ongoing heart failure despite LVAD implantation.

The patient received a heart from a donor succumbing to Methanol and presented persistent signs of AMR in the early phase (first two months) after transplant. In addition, signal-intense crescent-shaped structures were found in postoperative CMR scans, raising the suspicion of an imported cardiomyopathy.

Despite considerable advances, acute cellular and humoral rejection after allogeneic heart transplant represent a major threat to heart allografts. An imported cardiomyopathy is less common but remains a possible risk. Prior studies have found female gender and high immunization to be among the risk factors for rejection. The toxic effect of Methanol on the cardiovascular system is not well described, and severe heart failure after intoxication is only published in one single case report from 1987 (10). Historically, patients with intoxication have been considered as not being acceptable donors for transplant (11). However, since the demand for donors increased and waiting lists became longer, investigations focusing on poisoned donors have been made since the 1990s, and transplantation using the Methanol intoxicated donors has since been considered as feasible (11). The impact of Methanol on the human heart remains scarcely investigated in the literature. A study from 2014 focusing on electrocardiographic changes after poisoning reported clinical improvement once the Methanol intoxication had resolved (12). Also, intoxication may cause sinus tachycardia or T wave inversions, which represent a nonspecific sign of inflammatory response often observed after transplantation. Endomyocardial biopsy through femoral or jugular venous access remains the gold standard surveillance technique after transplant. Noninvasive imaging modalities including TTE and CMR allow more comprehensive monitoring with high availability and limited risk. In CMR, T1 and T2 relaxation times are in use to track ongoing rejection after heart transplant for two decades (13–16). More recently, a combination of T2 mapping times and ECV has been proposed as a diagnostic algorithm when rejection is suspected. At the time we performed the first CMR exam in our patient, we found slightly increased myocardial relaxation times and inflammatory changes in the T2-weighted images and after administration of Gadolinium. Myocardial edema and inflammation can be detected in both cellular (i.e. mediated by T-lymphocytes) and humoral (i.e. mediated trough B-cell derived antibodies) rejection. LGE quantifies replacement fibrosis and compares well with endomyocardial biopsies in the long-term FU. Moreover, LGE identifies different stages of cardiac allograft vasculopathy, including infarct patterns through myocardial scar detection (17). In our patient, the LGE distribution pattern was suspicious for mitochondriopathy (MELAS or MELAS-like) with cardiac involvement. The genetic study aimed to find a possible underlying cardiomyopathy, but the results did not yield in this direction. The two genetic variants found in the coding gene for Titin were rather unspecific (ACMG class 3). Titin mutations are usually involved in the pathogenesis of dilated cardiomyopathy and are estimated to be present in about a fifth of patients with severe forms (18). Mitochondrial genes have not been studied. Since their inheritance is essentially non-Mendelian, interpretation is highly speculative. For a transplanted patient, it would however have required myocardium to analyze mitochondrial DNA directly. Regarding the rejection, the genetic analysis was not focusing on transcripts known to be important in the context of AMR. The relevant transcripts reflecting a possible solid organ rejection include those of natural killer cells, macrophages, selective endothelial cells, interferon-gamma response as well as specific gene sets for AMR and heart tissue (4, 19). In a next step we could expand the scope of the investigation and perform whole-exome sequencing. In the case of our patient, a genetic peculiarity of the donor heart might explain in parts the macroscopic as well as the microscopic findings. Using the full spectrum of diagnostic tools is indispensable in the early management of allograft rejection after heart transplant.

## Conclusion

Our report exemplifies the complexity in the management of cardiac dysfunction in the early phase after transplant. Taking into consideration the large spectrum of possible underlying immune and non-immune related factors, acute rejection remains a major threat to heart allografts despite considerable advances in treatment. Our case highlights the need for awareness of donor intoxication in transplant recipients and the importance of recognizing the imitators of rejection. In this context, an imported cardiomyopathy is a less common, but still threatening finding. A combination of cardiovascular imaging, tissue sampling, and genetic sequencing could allow for the individualization of medical therapies and thus minimizing unnecessary interventions in these high-risk patients.

## Data Availability Statement

The raw data supporting the conclusions of this article will be made available by the authors, without undue reservation.

## Ethics Statement

Written informed consent was obtained from our patient for the publication of any potentially identifiable images or data included in this article.

## Author Contributions

LS wrote the main manuscript text. SK and FS contributed substantially to the revision of the text. KK provided histology images of the heart. HM performed the genetic analysis. All authors contributed to the article and approved the submitted version.

## Conflict of Interest

FS reports non-financial support from Medtronic, grants from Novartis, grants from Abbott, personal fees from Cardiorentis, outside the submitted work. CK and SK received support from the DZHK (German Center for Cardiovascular Research), Partner Site Berlin. SK received support from Philips Healthcare.

The remaining authors declare that the research was conducted in the absence of any commercial or financial relationships that could be construed as a potential conflict of interest.

## Abbreviations

ACMG, American College of Medical Genetics and Genomics; ATG, Antithymocyte globulin; CMR, Cardiac magnetic resonance tomography; DNA, Deoxyribonucleic acid; HLA, Human leukocyte antigen; ICU, Intensive care unit; IgG, Immunoglobulin G; IMC, Intermediate care unit; Intermacs, Interagency Registry for Mechanically Assisted Circulatory Support; ISHLT, International Society for Heart and Lung Transplantation criteria; LV, Left ventricle; LVEF, Left ventricular ejection fraction; LVAD, Left ventricular assist device; MELAS, Mitochondrial encephalomyopathy, lactic acidosis and stroke-like episodes; MMF, Mycophenolate Mofetil; nsVT, Non sustained ventricular tachycardia; OMIM, Online Mendelian inheritance of men; pAMR, Pathologic antibody-mediated rejection; TDI-Sm, Tissue Doppler imaging Systolic peak velocities (radial/longitudinal); RV, Right ventricle; RVEF, Right ventricular ejection fraction; Sm, Radial and Longitudinal Systolic peak velocity; TTE, Transthoracic echocardiography.
