# Supplementary material for: Case Report: Early Transplant Rejection of a Methanol-Intoxicated Donor Heart in a Young Female Patient. A Diagnostic Approach With CMR, Cardiac Biopsy, and Genetic Risk Assessment
Source: Front Immunol. 2021 Feb 22;11:575635. doi: 10.3389/fimmu.2020.575635 (PMC7938323; doi:10.3389/fimmu.2020.575635)
Supplement: Supplementary file 1 [file DataSheet_1.pdf]

Supplementary Material

Information on the genetic study of the donor DNA

A) Full list of investigated genes

We investigated the following genes involved in the most common cardiomyopathies:

ABCC9, ABCG5, ABCG, ACTA1, ACTA2, ACTC1, ACTN2, AKAP9, ALMS1, ANK2, ANKRD1, APOA4, APOA5, APOB, APOC2, APOE, BAG3, BRAF, CACNA1C, CACNAD1, CACNB2, CALM1, CALR3, CASQ2, CAV3, CBL, CBS, CETP, COL3A1, COL5A1, COL5A2, COX15, CREB3L3, CRELD1, CRYAB, CSRP3, CTF1, DES, DMD, DNAJC19, DOLK, DPP6, DSC2, DSG2, DSP, DTNA, EFEMP2, ELN, EMD, EYA4, FBN1, FNB2, FHL1, FHL2, FKRP, FKTN, FXN, GAA, GATAD1, GCKR, GJA5, GLA, GPD1L, GPIHBP1, HADHA, HCN4, HFE, HRAS, HSPB8, ILK, JAG1, JPH2, JUP, KCNA5, KCND3, KCNE1, KCNE2, KCNE3, KCNH2, KCNJ2, KCNJ5, KNJ8, KCNQ1, KLF10, KRAS, LAMA2, LAMA4, LAMP2, LDB3, LDLR, LDLRAP1, LMF1, LMNA, LPL, LTBP2, MAP2K1, MAP2K2, MIB1, MURC, MYBPC3, MYH11, MYH6, MYH7, MYL2, MYL3, MYLK, MYLK2, MYO6, MYOZ2, MYPN, NEXN, NKX2-5, NODAL, NOTCH1, NPPA, NRAS, PCSK9, PDLIM3, PKP2, PLN, PRDM16, PRKAG2, PRKAR1A, PTPN11, RAF1, RANGRF, RBM20, RYR1, RYR2, SALL4, SCN1B, SCN2B, SCN3B, SCN4B, SCN5A, SCO2, SDHA, SEPN1, SGCB, SGCD, SGCG, SHOC2, SLC25A4, SLC2A10, SMA3, SMAD4, SNTA1, SOS1, SREBF2, TAZ, TBX20, TBX3, TBX5, TCAP, TGFB2, TGFB3, TGFBR1, TGFBR2, TMEM43, TMPO, TNNC1, TNNI3, TNNT2, TPM1, TRDN, TRIM63, TRPM4 TTN, TTR, .TXNRD2, VCL, ZBTB17, ZHX3, ZIC3.

B) Information on Titin mutations

Two genetic variants in the coding gene for Titin with uncertain impact have been found in the analysis:

i.) TTN c.60500C>G, p.Thr20167Ser

The variant TTN p.Thr20167Ser results in an exchange of threonine to serin in the A-band region of the protein, affecting the cardiac isoforms N2B, N2BA, and the skeletal isoform N2A.

ii.) TTN c.83516G>A, p.Arg27839Gln, rs37682030

The variant TTN p.Arg27839Gln results in an exchange of arginine to glutamine in the A-band region of the protein affecting the cardiac isoforms N2B, N2BA, and the skeletal isoform N2A.

No other variants explaining neither the persistent rejection nor the cystic changes could be detected.

C) Limitations

Due to technical reasons, single nucleotides of the following genes have only been registered with 10-20 repetitions:

PRDM, SEPN1, NEXN, LMNA, ACTA1, ABCG5, COL3A1, DES, CRELD1, MYL3, FBN3, DPP6, TGFBR1, NOTCH1, MYPN, LDB3, BAG3, KCNQ1, MYBPC3, KCNJ5, CACNA1C, TBX3, LDBP2, FBN1, HCN4, LMF1, RANGRF, JUP, GAA, MAP2K2, SCN1B, RYR1, TRPM4, SNTA1, JPH2, SLC2A10.
